# High-Quality Draft Genomes from *Thermus caliditerrae* YIM 77777 and *T. tengchongensis* YIM 77401, Isolates from Tengchong, China

**DOI:** 10.1128/genomeA.00312-16

**Published:** 2016-04-28

**Authors:** Chrisabelle C. Mefferd, En-Min Zhou, Tian-Tian Yu, Hong Ming, Senthil K. Murugapiran, Marcel Huntemann, Alicia Clum, Manoj Pillay, Krishnaveni Palaniappan, Neha Varghese, Natalia Mikhailova, Dimitrios Stamatis, T. B. K. Reddy, Chew Yee Ngan, Chris Daum, Kecia Duffy, Nicole Shapiro, Victor Markowitz, Natalia Ivanova, Nikos Kyrpides, Amanda J. Williams, Tanja Woyke, Wen-Jun Li, Brian P. Hedlund

**Affiliations:** a School of Life Sciences, University of Nevada, Las Vegas, Las Vegas, Nevada, USA; b State Key Laboratory of Biocontrol and Guangdong Provincial Key Laboratory of Plant Resources, School of Life Sciences, Sun Yat-sen University, Guangzhou, China; c Key Laboratory of Microbial Diversity in Southwest China, Ministry of Education, Yunnan Institute of Microbiology, Yunnan University, Kunming, China; d Department of Energy Joint Genome Institute, Walnut Creek, California, USA; e SWCA Environmental Consultants, Las Vegas, Nevada, USA; f Nevada Institute of Personalized Medicine, University of Nevada, Las Vegas, Las Vegas, Nevada, USA

## Abstract

The draft genomes of *Thermus  tengchongensis* YIM 77401 and *T. caliditerrae* YIM 77777 are 2,562,314 and 2,218,114 bp and encode 2,726 and 2,305 predicted genes, respectively. Gene content and growth experiments demonstrate broad metabolic capacity, including starch hydrolysis, thiosulfate oxidation, arsenite oxidation, incomplete denitrification, and polysulfide reduction.

## GENOME ANNOUNCEMENT

Bacterial strains YIM 77401 and YIM 77777, members of the order *Thermales*, class *Deinococci*, were isolated from Frog Mouth Spring (Hamazui), Rehai National Park, Tengchong County, Yunnan Province, China (1). The draft genomes of the two strains were generated at the DOE Joint Genome Institute (JGI), Walnut Creek, California, USA, using Pacific Biosciences (PacBio) technology. A PacBio SMRTbell library was created and sequenced using the PacBio RS platform, which generated 191,522 filtered subreads totaling 522 Mbp for strain YIM 77401, and 280,439 filtered subreads totaling 626 Mbp for strain YIM 77777. HGAP version: 2.0.0 (2) was used to assemble raw reads. Genome annotation was performed using the JGI Prokaryotic Automatic Annotation Pipeline (3) with manual curation using GenePRIMP (4) and additional manual review using the Integrated Microbial Genomes–Expert Review (IMG-ER) platform (5). JGI’s library construction and sequencing protocols can be found at http://www.jgi.doe.gov.

The strain YIM 77401 genome encoded 2,726 predicted genes in 5 contigs, including 47 tRNA-encoding genes and 3 rRNA operons, and the strain YIM 77777 genome encoded 2,305 predicted genes in 4 contigs, including 50 tRNA-encoding genes and 3 rRNA operons. Both genomes included at least one megaplasmid (>100 kb), based on the presence of plasmid replicon domains (6). Analysis of carbohydrate-active enzymes (CAZymes) found in the strain YIM 77401 and YIM 77777 genomes revealed 39 and 32 CAZymes, respectively. Among these were 11 and 9 glycoside hydrolases (GHs) in strains YIM 77401 and YIM 77777, respectively, including GHs predicted for starch hydrolysis (GH13 and GH57) in both strains. This is consistent with amylase activity observed in both isolates. The genome of YIM 77401 featured genes involved in arsenite oxidation (*aioAB*), consistent with arsenite oxidation activity observed in this isolate. Both genomes contained a *sox* gene cluster composed of 10 genes (*soxABCDFVWXYZ*), predicted for thiosulfate oxidation (7), similar to other *Thermus* spp. (8–10); however, thiosulfate oxidation activity was only detected in YIM 77777.

Strain YIM 77401 contained a chromosomally encoded nitrate reductase gene cluster (*narGHJIK*) and two nitrate-nitrite transporters (*narK1* and *narK2*), similar to other *Thermus* spp. (9). Genes encoding the catalytic subunit of a *cd*-cytochrome nitrite reductase (*nirS*) and nitric oxide reductase (*norBC*) were also found in this genome. However, nitrous oxide reductase (*nos*) genes, which catalyze the reduction of nitrous oxide to dinitrogen, were absent, consistent with the incomplete denitrification phenotype found in several *Thermus* spp. (9, 11) and the production of N_2_O as the terminal denitrification product by YIM 77401. Additionally, YIM 77401 and YIM 77777 contained genes for polysulfide reduction (*psrABC*), which is similar to other *Thermus* genomes (9) and consistent with polysulfide reductase activity in both isolates.

### Nucleotide sequence accession numbers.

These whole-genome shotgun projects have been deposited in GenBank under accession numbers JQNC01000001 to JQNC01000004 (YIM77777) and JQLK01000001 to JQLK01000005 (YIM77401). The genome sequence is available from GenBank (NZ_JQNC00000000; GI: 740207912) for *Thermus caliditerrae* YIM77777, and from GenBank (NZ_JQLK00000000; GI:740202250) for *T. tengchongensis* YIM77401. The data are also available at the Joint Genome Institute (JGI) Integrated Microbial Genomes (IMG) system (12).
